# A Preliminary Study: N-acetyl-L-cysteine Improves Semen Quality following Varicocelectomy

**DOI:** 10.22074/ijfs.2016.4777

**Published:** 2016-04-05

**Authors:** Foroogh Barekat, Marziyeh Tavalaee, Mohammad Reza Deemeh, Mahsa Bahreinian, Leila Azadi, Homayoun Abbasi, Shahla Rozbahani, Mohammad Hossein Nasr-Esfahani

**Affiliations:** 1 Department of Reproductive Biotechnology, Reproductive Biomedicine Research Center, Royan Institute for Biotechnology, ACECR, Isfahan, Iran; 2 Department of Biology, Flavarjan Branch, Islamic Azad University, Flavarjan, Isfahan, Iran; 3 Isfahan Fertility and Infertility Center, Isfahan, Iran

**Keywords:** DNA Fragmentation, Protamines, Oxidative Stress, Varicocele, NAC

## Abstract

**Background:**

Surgery is considered the primary treatment for male infertility from clinical varicocele. One of the main events associated with varicocele is excessive production of reactive oxygen species (ROS). N-acetyl-L-cysteine (NAC), an antioxidant that scavenges free radicals, is considered a supplement to alleviate glutathione (GSH) depletion during oxidative stress. Despite beneficial effects of NAC in other pathological events, there is no report on the effect of NAC in individuals with varicocele. Therefore, the aim of this study is to evaluate the outcome of NAC on semen quality, protamine content, DNA damage, oxidative stress and fertility following varicocelectomy.

**Materials and Methods:**

This prospective clinical trial included 35 infertile men with varicocele randomly divided into control (n=20) and NAC (n=15) groups. We assessed semen parameters, protamine content [chromomycin A3 (CMA3)], DNA integrity [terminal deoxynucleotidyltransferase-mediated dUTP nick-end labeling (TUNEL)] and oxidative stress [2', 7'-dichlorodihydrofluorescein-diacetate (DCFH-DA)] before and three months after varicocelectomy.

**Results:**

Percentage of abnormal semen parameters, protamine deficiency, DNA fragmentation and oxidative stress were significantly decreased in both groups compared to before surgery. We calculated the percentage of improvement in these parameters compared to before surgery for each group, then compared the results between the groups. Only percentage of protamine deficiency and DNA fragmentation significantly differed between the NAC and control groups.

**Conclusion:**

The results of this study, for the first time, revealed that NAC improved chromatin integrity and pregnancy rate when administered as adjunct therapy post-varico- celectomy (Registeration Number: IRCT201508177223N5).

## Introduction

Production, maturation, and transport of sperm occur in the male reproductive tract (1). Molecular and structural anomalies in this system may result in male infertility (2). The most common structural anomaly associated with the reproductive tract is abnormal enlargement of the pampiniform plexus of veins within the scrotum, commonly referred to as varicocele (3). Although the association between male infertility and varicocele has been known since the past century, a limited number of studies exist that report the molecular and genetic bases of varicocele. Therefore, further research in this field may open new strategies for treatment of male infertility due to varicocele (4).

One of the key events in the pathology of varicocele is excessive production of reactive oxygen species (ROS) (5). Oxidative stress results from an imbalance between ROS production and antioxidant capacity (6). However, it is important to bear in mind that ROS acts as a doubleedged sword. Although it serves as a key signal molecule in physiological processes, ROS also has a role in pathological processes (7). In pathological conditions, two roles have been envisaged for overproduction of ROS: i. ROS induced damage to sperm membrane reduces sperm motility and ability of the sperm to fuse with the oocyte, and ii. ROS directly damages sperm DNA and subsequently effects genomic integrity of the embryo (8, 9). Therefore, antioxidant therapy may overcome the deleterious effects of ROS in individuals with varicocele (10). Varicocelectomy, especially through microsurgery, has been shown to restore testicular volumes and semen parameters, as well as reduce the degree of DNA fragmentation (11, 12). Despite these beneficial effects of varicocelectomy, fewer studies have focused on the role of antioxidants as adjunct therapy along with varicocelectomy (13).

N-acetyl-L-cysteine (NAC) is a derivative of the naturally occurring amino acid L-cysteine that has free radical scavenging activity. Therefore, it is supplemented to alleviate glutathione (GSH) depletion during oxidative stress (14, 15).

Previous studies have shown both *in vivo* (6) and *in vitro* (16, 17) addition of NAC may improve semen parameters and thereby improve male fertility. Despite these beneficial effects of NAC, there has been no report on the effect of NAC in individuals with varicocele. Therefore, the aim of this study is to evaluate the effects of NAC on semen quality, protamine content, DNA damage, oxidative stress and fertility following microsurgical varicocelectomy.

## Materials and Methods

This prospective clinical trial carried out between 2011 and 2013, was approved by the Ethics Committee for Research Involving Human Subjects at Royan Institute and the Isfahan Fertility and Infertility Center. All individuals gave informed consent prior to participation in the study.

### Inclusion and exclusion criteria

Inclusion criteria included male gender, age younger than 45 years, primary infertility, and left-sided varicocele (grades II and III) diagnosed by palpation and Doppler duplex ultrasound. Exclusion criteria included grade I varicocele, azoospermia, recurrent varicocele, leukocytospermia, urogenital infections, testicular size discrepancy, abnormal hormonal profile, anatomical disorders, Klinefelter’s syndrome, cancer, fever in the 90 days prior to surgery, seminal sperm antibodies, excessive alcohol and drug consumption, previous history of scrotal trauma or surgery, and occupational exposure.

We included female partners who were less than 35 years of age that had normal ovulatory cycles and patent tubes (hysterosalpingography or laparoscopy) in this study. Individuals with endometriosis, cycle irregularity, or gross anatomical abnormalities were excluded.

### Patient selection

This study was designed similar to a blinded clinical trial. A total of 40 individuals with grades II and III varicocele enrolled in this study. Following microsurgery, the patients were randomly allocated to the control or treatment groups. In the control group, individuals received no drug after varicocelectomy (n=20). In the treatment or NAC group (n=15), the individuals received three tablets of NAC (200 mg daily) post-varicocelectomy for three months based on a previous study (6). In this study, five individuals were excluded from the treatment group due to lack of compliance with NAC use, according to the study protocol. All parameters assessed in this study were carried out by a single trained individual unaware of treatment assignment. Duration of infertility was 2.1 ± 0.2 years and duration of marriage was 3.8 ± 0.3 years in individuals with varicocele.

We initially aimed to include a group in which the individuals did not want to undergo surgery, as either a control (without surgery) or treatment (without surgery+NAC) group. However, there were few individuals that refused to undergo surgery since the majority of these individuals had referred for infertility treatment.

Prior to surgery and at three months post-surgery, all participants provided semen samples by masturbation after 3-4 days of abstinence. Semen samples were analyzed according to World Health Organization (WHO) criteria (18). After immobilizing the sperm with a fixing solution, we evaluated the sperm concentration by a Makler counting chamber. Sperm were expressed as million/ml. Sperm motility and morphology were assessed by the Computer Aided Sperm Analysis (CASA) system (LABOMED, SDC313B). Sperm morphology was evaluated by Diff-Quik staining. DNA fragmentation and protamine deficiency were assessed with the TUNEL assay (19) and chromomycin A3 (CMA3) staining (20).

### Assessment of sperm morphology (Diff-Quik staining)

A sperm suspension (20-30 μl) was smeared on the slide, allowed to air dry and stained with the prepared kit (18). Briefly, slides were immersed for 30 seconds into methanol (fixative), eosin (stain basic proteins) and a thiazin-like stain (stain DNA), respectively. Subsequently, slides were dipped into water to remove excess dye and allowed to air dry. We evaluated 200 sperm per slide.

### Assessment of DNA fragmentation and protamine deficiency sperm by TUNEL and CMA3 staining

DNA fragmentation was evaluated with the aid of a terminal deoxynucleotidyltransferasemediated dUTP nick-end labeling (TUNEL) kit (Apoptosis Detection System Fluorescein, G3250, Promega, Mannheim, Germany) according to Kheirollahi-Kouhestani et al. (19). On each slide, 500 sperm were assessed under an epifluorescent microscope (BX51, Olympus, Japan) at ×100 magnification. Sperm with red heads were considered to have intact DNA. Those with green heads were considered to have fragmented DNA.

The percentage of sperm with protamine deficiency was assessed with CMA3 staining, according to Nasr-Esfahani et al. (20). On each slide, we assessed 500 sperm under an epifluorescent microscope (BX51, Olympus, Japan) at ×100 magnifications. Those sperm that stained light yellow were considered as CMA3 positive or protamine deficient; however, sperm that stained dark yellow were considered to have normal protamine content.

### Assessment of reactive oxidative species by DCFH-DA

ROS status was assessed using a 2', 7'-dichlorodihydrofluorescein- diacetate (DCFH-DA, D6883, Sigma Co., USA) probe according to Kiani-Esfahani et al. (21). Briefly, a 2.5 mM stock solution of H2DCF-DA was prepared in dimethyl sulfoxide and stored at -70°C. A total of one million sperm were treated with 5 μM H2DCFDA for 30 minutes, while percentages of ROS positive sperm were defined by flow cytometery.

### Statistical analysis

The Kolmogorov-Smirnov Z test was used to assess normal data distribution. The student’s t test was carried out using the Statistical Package for the Social Studies (SPSS11.5, Chicago, IL, USA). For comparison between control and NAC groups, we used the independent t test. For comparison between the preand post-surgery in control and NAC groups, the paired t test was used. The differences with values of P<0.05 were considered statistically significant.

## Results

The study population consisted of 35 individuals with grades II and III varicocele. The mean ages of male participants was 30.1 ± 4.4 (range: 22-45) years; for females, it was 26.6 ± 4.9 (range: 17-35) years. During this study, individuals with varicocele were randomly assigned to control (non-NAC) and NAC groups. In order to show that there were no significant differences between the two groups before treatments, the sperm parameters between control and NAC groups were compared. The results showed no significant difference between the two groups (Table 1). Age range of females (27.4 ± 5.7 vs. 26.05 ± 4.2 years) and males (30.7 ± 1.4 vs. 29.6 ± 0.7 years) were also similar between the two control and NAC groups.

**Table 1:** Comparison of pre-surgery semen parameters and ages in men with varicocele in the control and N-acetyl-L-cysteine (NAC) groups


Parameters	NAC group (n=15)	Control group n=20)

Concentration (106/ml)	23.94 ± 5.9	29.7 ± 6.9
Sperm motility (%)	36.94 ± 5.5	34.92 ± 4.68
Abnormal morphology (%)	99.28 ± 0.2	99.3 ± 0.16
Volume (ml)	3.9 ± 0.5	3.47 ± 0.36
Age (Y)	30.73 ± 1.4	29.64 ± 0.74


### Comparison of sperm parameters before and after surgery in control and NAC groups

**Fig.1:**
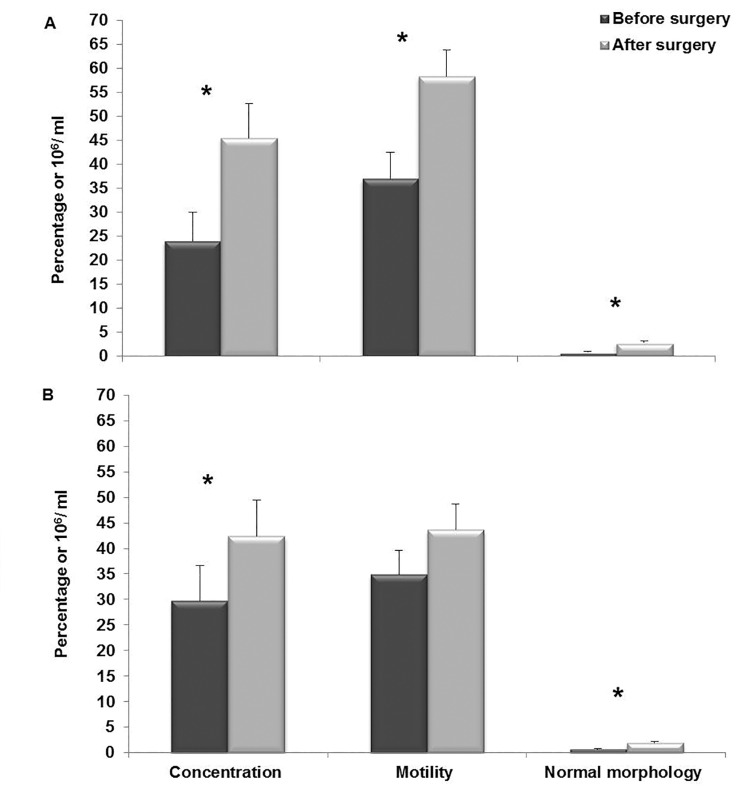
Comparison of sperm parameters before and after surgery in A. N-acetyl-L-Cysteine (NAC) and B. Control group. *; Indicate significant difference before and after surgery.

In the NAC group, sperm concentration prior to surgery (23.9 ± 5.9) compared to after surgery (45.4 ± 7.1, P<0.01), percentage of sperm motility prior to surgery (36.9 ± 5.5) versus after surgery (58.2 ± 5.4, P<0.01) and normal morphology prior to surgery (0.71 ± 0.1) versus after surgery (2.71 ± 0.3, P<0.01), showed significant improvement (Fig .1A). Similarly, in the control group, sperm concentration (29.7 ± 6.9 vs. 42.4 ± 7.02, P<0.05) and normal morphology (0.7 ± 0.1 vs. 1.9 ± 0.2, P<0.01) also significantly improved following surgery. Unlike the NAC group, however, the percentage of sperm motility (34.9 ± 4.6 vs. 43.6 ± 4.9, P=0.1) did not increase following surgery in the control group (Fig .1B).

### Comparison of protamine content, DNA integrity, and reactive oxygen species status before and after surgery between control and NAC groups

**Fig.2:**
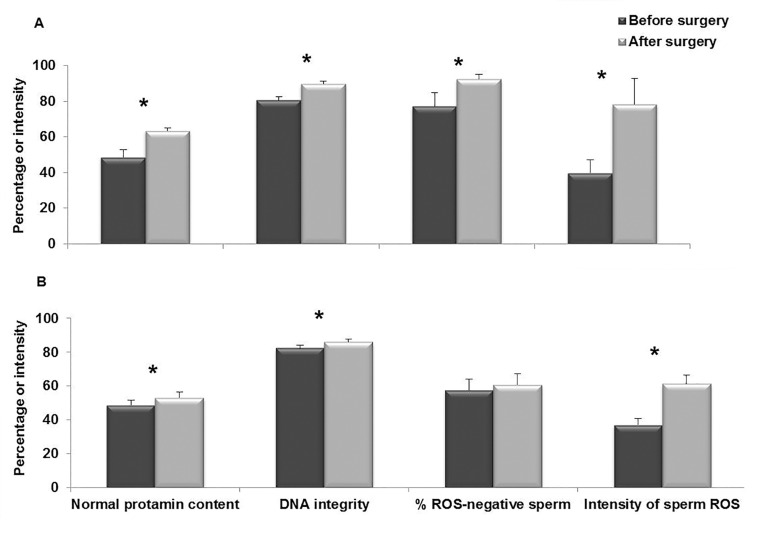
Comparison of different parameters before and after surgery in A. N-acetyl-L-cysteine (NAC) and B. Control groups. *; Indicate significant difference before and after surgery and ROS; Reactive oxygen species.

In the NAC group, percentages of normal protamine content (48.7 ± 4.1 vs. 63.5 ± 1.6, P<0.01), percentages of DNA integrity (80.6 ± 1.8 vs. 89.8 ± 1.4, P<0.01), percentage of ROS-negative sperm (77.2 ± 7.5 vs. 92.3 ± 2.6, P<0.05), and intensity of sperm ROS (40.02 ± 7.1 vs.78.1 ± 14.6, P<0.01) significantly increased following surgery (Fig .2A). Similarly, in the control group, percentages of normal protamine content (48.7 ± 2.9 vs. 53.2 ± 3.1, P<0.01), percentages of DNA integrity (82.2 ± 1.7 vs. 85.9 ± 1.7, P<0.01) and intensity of sperm ROS (37.2 ± 3.6 vs. 61.3 ± 5.3, P<0.01) also increased significantly following surgery. Unlike the NAC group, the percentage of ROS-negative sperm (57.6 ± 6.6 vs. 60.9 ± 6.4, P=0.3) did not increase following surgery in the control group (Fig .2B).

### Comparison of percentage of improvement of sperm parameters, status of chromatin and reactive oxygen species between NAC and control groups

In order to evaluate improvement in these parameters between the NAC and control groups, we calculated the difference between the mean values of these parameters before and after surgery, divided by the mean values of these parameters before surgery, times 100. There was no significant difference in improvement of sperm concentration (220.5 ± 75.9 vs. 149.7 ± 81.3, P=0.5), percentage of sperm motility (100.4 ± 29.5 vs. 44.6 ± 21.8, P=0.1), abnormal morphology (100 ± 16.3 vs. 81.8 ± 27.1, P=0.5), percentage of ROS-negative sperm (36.6 ± 23.1 vs. 16.5 ± 9.5, P=0.3) and intensity of sperm ROS (76.6 ± 29.5 vs. 64.9 ± 19.5, P=0.7) observed between the NAC and control groups, respectively (Figes.3, 4). However, we found significant differences in percentages of improvement of normal protamine content (45.6 ± 13.5 vs. 11.9 ± 4.7, P<0.05) and DNA integrity (11.8 ± 2.01 vs. 4.7 ± 1.3, P<0.01) between the NAC and control groups, respectively (Fig .4).

**Fig.3:**
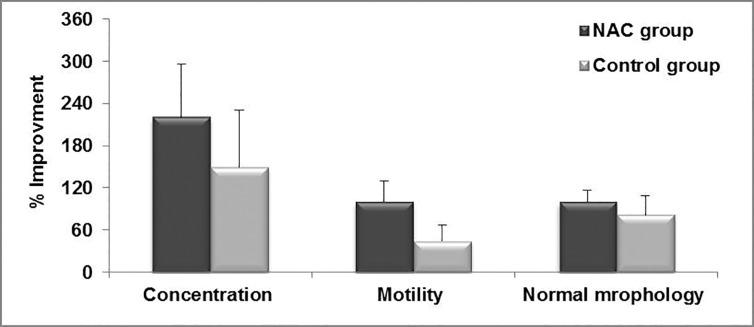
Comparison of percentage of improvement in semen parameters in N-acetyl-L-cysteine (NAC) and control groups.

**Fig.4:**
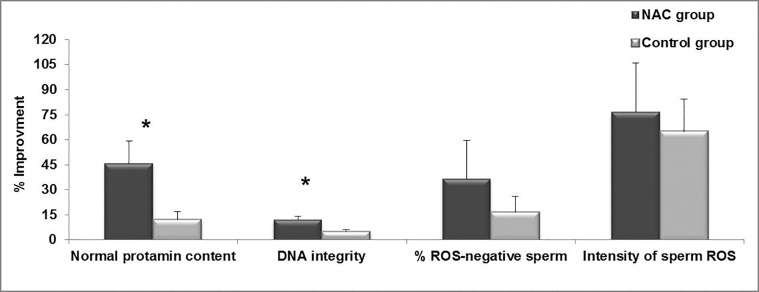
Comparison of percentage improvement in different parameters in N-acetyl-L-cysteine (NAC) and control groups. *; Indicate significant difference between NAC and control groups and ROS; Reactive oxygen species.

### Clinical pregnancy

The percentage of clinical pregnancy in the NAC group was 33.4% (5/15), in the control group, this result was 10% (2/20).

## Discussion

Oxidative stress induced by heat stress is considered the central element that contributes to the etiology of infertility in individuals with varicocele (22). Therefore, surgical varicocele repair is expected to be beneficial to these individuals by alleviating heat, and thereby oxidative stress (10). A second approach to alleviate the varicocele associated ROS is antioxidant therapy (10, 13). In order to improve the efficiency of surgical treatment, concomitant therapy with antioxidants has be e n suggested (13).

We aimed to evaluate the effect of NAC on semen quality, protamine content, DNA damage, oxidative stress and fertility following varicocelectomy, as well as to compare these parameters in individuals with varicocele who did not use NAC after surgery. NAC is one of the oldest and most powerful antioxidants that treat various diseases, including respiratory disorders, heart disease, heavy metal poisoning, overdose with acetaminophen and epilepsy (6, 23).

The results of this study showed that sperm parameters (concentration, motility and normal morphology) significantly improved after surgery compared to before surgery in both the NAC and control groups, with the exception of the percentage sperm motility which insignificantly improved in the control group. Despite controversies on the degree of improvement of each semen parameters post-varicocelectomy (24), this insignificant improvement of motility in the control group was consistent with previous reports (25). However, the results of this study suggested that NAC might have an additional value by improving sperm motility post-varicocelectomy. In contrast to these results, Comhaire et al. (26) reported that although NAC improved sperm concentration and acrosome reaction, it had no effect on motility and morphology.

Despite the importance of semen parameters in fertility, many researchers have suggested that other sperm function characteristics should be considered along with these parameters when assessing fertility (27, 28). Therefore, in this study, we have assessed genomic integrity and ROS production.

Sperm DNA becomes susceptible to damage by three postulated routes: i. Improper packaging of DNA during spermiogenesis, ii. Oxidative stress and iii. Apoptosis (29). We have assessed DNA damage, ROS production and sperm nuclear maturity before and after surgery in the NAC and control groups. Of note, all parameters improved after surgery in both groups, except for the percentage of ROS negative sperm in the control group. The percentage of sperm motility did not significantly improve before and after surgery in the control group. Therefore, this lack of significant improvement in motility post-surgery in the control group might be related to the lack of significant improvement in percentage of ROS negative sperm. In addition, the improved sperm motility in the NAC group was associated with a reduced percentage of sperm producing ROS or increased percentage of ROS negative sperm, which might be related to NAC treatment. The existence of this correlation might be explained by the cascade of events that begin with ROS associated with lipid peroxidation (30), which in turn, reduces membrane fluidity. This prevents axonemal protein phosphorylation and leads to sperm immobilization (30, 31). NAC may break these chains of events.

In this study, there was higher mean ROS intensity in semen samples after surgery compared to before surgery. This contrasted expectations since ROS production should decrease post-surgery. This was likely due to leakage of ROS or reduced production of ROS after loss of enzymes in the sperm of these individuals, which were in their final stage of apoptosis. This supposition has been previously presented by Aitken et al. (32) who reported that initially ROS positive sperm progressively become TUNEL positive. This indicated that in individuals with varicocele, despite higher production of ROS, the ROS might leak from these cells or sperm in the final stage of apoptosis, hence the enzymes that produced ROS were not as efficient. This might account for the reduced intensity of ROS.

In order to further differentiate between the role of surgery and antioxidant therapy, we calculated the percentage of improvement relative to before surgery for sperm parameters, sperm protamine content, DNA integrity, and ROS in each group and compared them between the NAC and control groups. The percentage of improvement was calculated by the difference between the mean values of a parameter before and after surgery divided by its initial value before surgery. The results revealed no significant difference for percentage of improvement for the semen parameters between the NAC and control groups. However, among the sperm functional parameters assessed, the percentage of improvement for the normal protamine content and DNA fragmentation significantly differed between the NAC and control groups, despite no initial difference between the two groups before surgery. These results suggested that despite the similar process of surgery in the two groups, the difference between percentages of improvement in the two groups was due to antioxidant activity of NAC. Possibly other etiological factors might account for this difference, which were improved by NAC, or NAC might overcome the secondary side effects of surgery. However, the role of NAC on its own in treatment of varicocele has yet to be elucidated.

Despite the higher rate of pregnancy in the NAC group compared to the control group, we did not compare clinical pregnancy rates between the groups. Due to the limited number of cases, further study would be warranted.

## Conclusion

NAC can scavenge free radicals, increase GSH production and reduce disulfide bonds, as well as viscosity and elasticity of semen, which are important for fertility. This, in conjunction with the results of the current study (improved sperm parameters, DNA integrity and chromatin packaging), may account for the higher pregnancy rate in NAC group. In order to reach this conclusion, additional studies are recommended.
